# Supplementary material for: Shallow-angle needle guide for ultrasound-guided internal jugular venous catheterization: A randomized controlled crossover simulation study (CONSORT)
Source: PLoS One. 2020 Jun 30;15(6):e0235519. doi: 10.1371/journal.pone.0235519 (PMC7326219; doi:10.1371/journal.pone.0235519)
Supplement: S2 File — (PDF) [file pone.0235519.s003.pdf]

中心静脈穿刺合併症の少ない Shallow angle ニードルガイドの開発（シミュレーション研究）

研究実施計画書
第 1 版（平成 30 年 5 月 22 日）

研究代表者：杏林大学医学部麻酔科学　教授
徳嶺　譲芳

事務局
杏林大学医学部麻酔科学教室　医局
渡辺　邦太郎
〒181-8611 東京都三鷹市新川 6-20-2
Tel：　0422-47-5511
Fax：0422-43-1504
E-mail:kunitarowatanabe@gmail.com

1．研究の概要
1.1 背景・目的
超音波ガイドを用いた中心静脈穿刺は、中心静脈穿刺の標準手技となった。これは、超音波ガイド下中心静脈穿刺（超音波ガイド法）が、従来の盲目的な穿刺方法と比べ、成功率が高く、合併症が低いというエビデンスに基づく。しかし、平成 29 年 3 月に日本医療安全調査機構による提言では、中心静脈穿刺の死亡事故の約半数が超音波ガイド法であった。エビデンスと乖離するこの現実は、術者の稚拙な手技と超音波に特有のピットフォールに陥ったことが原因であると考えられる。
手技を補うため、ニードルガイドが開発・市販されている。しかし、市販のニードルガイドは、角度が固定しているため使い難く普及していない。超音波画像上で針をナビゲーションするシステムを研究中に、合併症の発生は、静脈に対する穿刺角度が関係しているという発想を得た。本研究は、この発想を基に穿刺角度の浅い鈍角穿刺ニードルガイドを作り、内頚静脈穿刺の安全に寄与することにある。
従来のニードルガイドが穿刺の成功率を上げることを目的としているのに比べ、新しいニードルガイドは、合併症を防ぐことを目的としている。今まで、「穿刺の成功率が上がれば合併症の発生が減る」と考えらえてきたが、現実には成功率が上がっても、合併症は依然として発生している。それは、合併症の原因となっている超音波画像が引き起こすピットフォールに対処する方法が検討されていなかったからである。

1.2 シェーマ

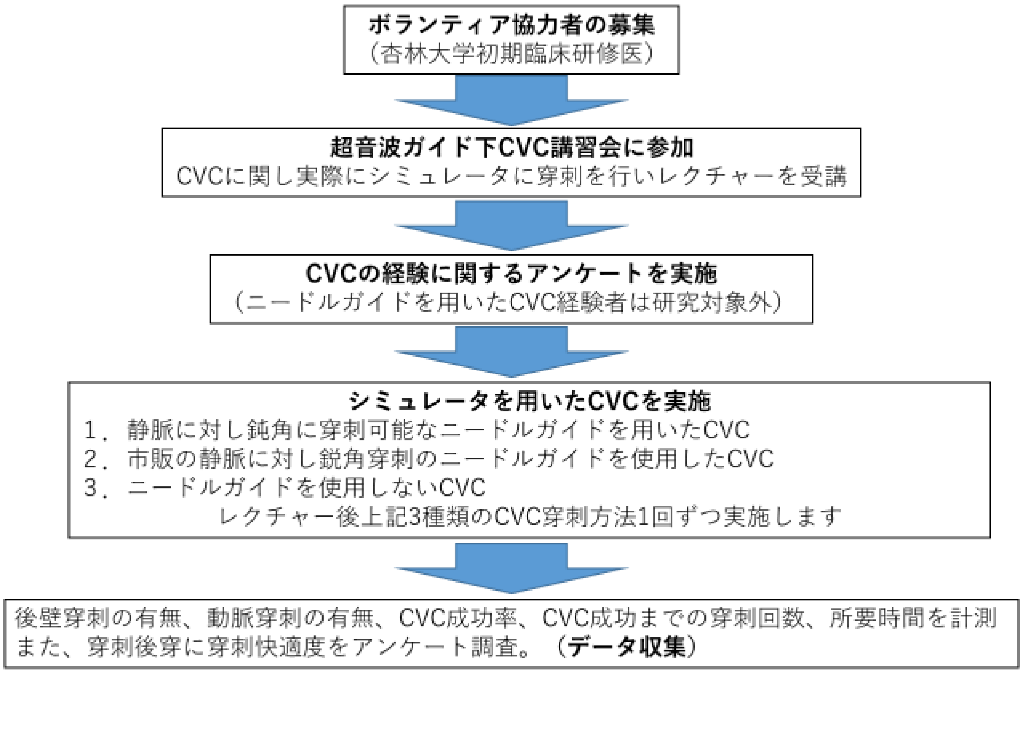

2．研究背景

近年、中心静脈カテーテル挿入を安全に施行するために超音波ガイド下に行うことが一般化しつつある。当院においても中心静脈穿刺に関する医療安全講習会を受講し、超音波ガイド下中心静脈穿刺のトレーニングを行った上で中心静脈穿刺のライセンスを交付している。超音波画像診断装置を用いることで、内頚静脈や総頚動脈の位置を直接確認しながら穿刺することが可能である。これまでのランドマーク法に比べ成功率が高く、合併症発生率が低い(1)。しかし、超音波ガイド下中心静脈穿刺の際に必要な穿刺針の画像描出は、十分なトレーニングが必要である。その技能が十分でないと重大な合併症が発生するおそれがある。近年探触子にニードルガイド装置を固定する機械式ニードルガイドが開発され、穿刺針を簡便に描出する方法開発が模索されている。そのような中で、穿刺針の磁場を感知し針をナビゲーションする磁性式ニードルガイド機能付きの超音波画像診断装置が開発された(2)。これまでのニードルガイドを用いない超音波ガイド下中心静脈穿刺に比べて磁性式ニードルガイドを用いた超音波ガイド下中心静脈穿刺は高い成功率を示している(3)。しかし、我々の研究では磁性式ニードルガイドを用いて穿刺を行っても合併症発生率（後壁穿刺率）は減少しなかった。合併症の発生は、静脈に対する穿刺角度が関係していることが明らかとなった。従来の穿刺法は静脈へ鋭角に刺入する従来の穿刺方法では針先のわずかな動きにより後壁穿刺が生じ、磁性式ニードルガイドはその危険を減少させなかった。磁性式ニードルガイドを用いて静脈に対し鈍角穿刺を行ったところ後壁穿刺率の著名な減少を認めた。磁性式ニードルガイド機能付き超音波診断装置は、一般的に普及しておらず国内では販売の目途もない。

3.目的

既存の超音波診断装置に着脱可能な鈍角穿刺可能なニードルガイドの開発と有用性の検証が本研究目的である。今回我々が開発した静脈に対し鈍角に穿刺可能なニードルガイドを用いたCVC、現在市販されている静脈に対し鋭角穿刺のニードルガイドを使用したCVC、ニードルガイドを使用しないCVCのそれぞれの後壁穿刺率を、CVC穿刺用シミュレータを用いて比較する。

4.研究対象

当院当科研修中の初期臨床研修医を対象とする。
当院当科主催の超音波ガイド下CVC講習会で研究への協力を呼びかける。
参加者背景として、中心静脈穿刺の経験の有無、経験症例数、超音波ガイド下中心静脈穿刺の経験の有無、経験症例数、超音波診断装置の使用経験の有無、当院における中心静脈穿刺ライセンスの有無をアンケート調査する。
除外対象として、ニードルガイドを用いた超音波ガイド下中心静脈穿刺を経験したことのある者とする（CVC講習会開催時にアンケートを行う、別紙参加者登録票参照）。

5.研究方法

以下の3種類の穿刺方法の比較を行う。

１．静脈に対し鈍角に穿刺可能なニードルガイドを用いたCVC（方法1）
２．市販の静脈に対し鋭角穿刺のニードルガイドを使用したCVC（方法2）

３．ニードルガイドを使用しない CVC（方法 3）

協力者はすべての穿刺方法のレクチャーを受けた後に、中心静脈穿刺用シミュレータに穿刺を行う。協力者はすべての穿刺方法を実施するクロスオーバー比較試験である。穿刺方法の順番は乱数表にて、無作為に行う。評価者はシミュレータ血管内に内蔵されているビデオカメラ画像を観察し下記評価項目を記録する。

6.評価項目
主要評価項目は、内頸静脈後壁穿刺率である。

副次的評価項目は、中心静脈穿刺成功率、総頚動脈穿刺率、穿刺成功するまでの穿刺回数、穿刺成功までの所要時間である（探触子を手に取ってから穿刺針が内頸静脈穿刺しガイドワイヤー挿入完了まで）。
また穿刺時の快適度をアンケート調査する（Likert scale を用いる、1 点：全く穿刺しにくい、5 点：非常に穿刺しやすい。1-5 点の 5 段階評価）。また最も好ましいと考える穿刺方法をアンケート調査する（別紙穿刺結果記録用紙参照）

7.目標協力者数
目標協力者数を 50 名とする。
根拠は、我々の過去の研究から設定した。中心静脈穿刺用シミュレータを用いて磁性式ニードルガイドを用いて従来の静脈に対し鋭角穿刺法と鈍角穿刺法による後壁穿刺率の比較を行った。
その結果は従来の鋭角穿刺法で 41%、鈍角穿刺で 9%であった。この結果をもとに、我々の開発した鈍角穿刺ニードルガイドの後壁穿刺率を 10%程度と仮定し、検出力 80%、αエラー5%として計算した必要症例数は 34 名であった。除外者を考慮し 50 名とした。

8.シミュレータ、穿刺環境、機材の役割
中心静脈穿刺用シミュレータの内頸静脈圧は 10mmHg、総頚動脈圧は 50mmHg に調整する。協力者は 1 回穿刺するごとにウォッシュアウト期間として 15 分以上間隔を空けてから次の穿刺を行う（休憩時間や昼食時間を利用する）。
中心静脈穿刺用シミュレータの内頸静脈にはビデオカメラを内蔵しており、血管内での穿刺針の動きを観察し記録する。また穿刺時の超音波動画も記録する。

9. 必要機材
① 静脈内のファイバー内視鏡ビデオシステム（当教室所蔵）。
② 市販のニードルガイドは Civco 社製 AccuSITE を使用（当教室所蔵）。
③ 静脈に対し鈍角に穿刺可能なニードルガイド（当教室所蔵）。
CAD ソフト（３DBuider : Microsoft 社）を用いて設計を行い３D プリンターで出力（DMM make 社）し作成を行う（図 1，2 参照）。

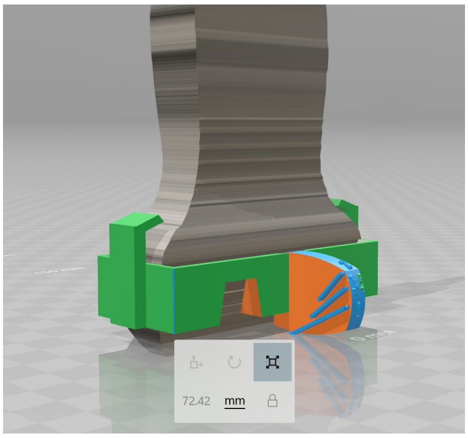

図１． ニードルガイド３D 画像

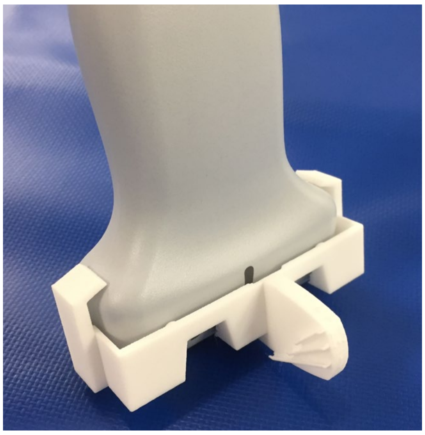

図 2． 出力したニードルガイド

④ 超音波診断装置：SonoSite 社 EDGE、プローベは HFL50、HFL38 を使用する（当院所蔵）。
⑤ シミュレータ：本体（当教室所蔵）

10．研究の実施期間：倫理員会承認後～2020 年 8 月 31 日

11．　研究が行われる機関または実施場所：杏林大学医学部付属病院中央手術室、シミュレーションセンター。

12．予想される結果：従来型の鋭角穿刺ニードルガイドを用いた超音波ガイド下中心静脈穿刺と比べ、我々の開発した鈍角穿刺ニードルガイドを用いた超音波ガイド下中心静脈穿刺の方が、後壁穿刺率が有意に低下することが予想される。

13．結果の匿名化：結果記録用紙には研修医氏名は記載せず、被験者番号を割り当て、対応表を別紙作成し管理する。

14．研究における倫理的配慮について：本研究はシミュレータを用いた研究であり実際に患者に対する臨床研究ではない。ただしボランティアで協力する研修医は自由参加であることを伝えた上で参加を呼びかけ、口頭および文書による同意を得る。研究情報を公開し途中辞退も可能とする（別紙超音波ガイド下中心静脈穿刺講習会を受講される先生方へ参照）。
参加報酬は、中心静脈穿刺の穿刺トレーニングを無償で受けられることである。

15．経費
① 市販ニードルガイド用ブラケット（3000 円/個）12 個購入。
② ３D プリント出力（3000 円/個）試作器を含め 10 個。
③ 交換用シミュレータパット（9000 円/個）：100 回程度の穿刺ごとに交換。10 個程度購入予定。

16．研究体制

研究責任者：麻酔科学・助教（任期制）（大学院生）・渡辺邦太郎
研究代表者：麻酔科学・教授・德嶺譲芳
研究分担者：麻酔科学・講師・森山久美

17. 参考文献

1. Brass P, Hellmich M, Kolodziej L, Schick G, Smith AF. Ultrasound guidance versus anatomical landmarks for internal jugular vein catheterization. Cochrane Database Syst Rev. 2015;1:CD006962. doi: 10.1002/14651858.CD006962.pub2.

2. Gadsden J, Latmore M, Levine DM. Evaluation of the eZono 4000 with eZGuide for ultrasound-guided procedures. Expert Rev Med Devices. 2015 ;12:251-61. doi: 10.1586/17434440.2015.995095. Epub 2014 Dec 29.

3. Auyong DB, Yuan SC, Rymer AN, Green CL, Hanson NA. A randomized crossover study comparing a novel needle guidance technology for simulated internal jugular vein cannulation. Anesthesiology. 2015;123:535-41. doi: 10.1097/ALN.0000000000000759.

Development of a shallow angle needle guide to prevent mechanical complications during central venous catheterization (A simulation study)

Study protocol

1st edition, May 22, 2018

Research director
Joho Tokumine MD, PhD
Clinical Professor

Principal investigator
Kunitaro Watanabe
Assistant Professor
Department of Anesthesiology
Kyorin University School of Medicine
E-mail:kunitarowatanabe@gmail.com

Office
Department of Anesthesiology,
Kyorin University School of Medicine
6-20-2 Shinkawa, Mitaka City, Tokyo 181-8611, Japan
Tel:0422-47-5511, Fax0422-43-1504

1. Research Outline

1.1 Background・Objectives

Ultrasound guidance during central venous catheterization is associated with high success rates and low mechanical complication rates and is recognized as the "gold standard" technique. The *Japan Medical Safety Research Organization* reported "*Analysis of deaths related to the complications of Central Venous Catheterization (2017)*". In this report, about half of the procedures were performed using ultrasound-guidance. The expert committee commented "Real-time ultrasound-guided technique has a pitfall that could lead to serious complications. The operator should receive simulator training in advance".

Needle guides have been developed to assist operators during ultrasound guided venous catheterization. Commercially available needle guides are still not popular because of difficultly in handling with a fixed needle holder. A previous study of a needle navigation system showed that a shallow angle of approach of the needle may reduce the incidence of posterior vein wall injuries. We postulated that the mechanical complication rate may be related to the needle's angle of approach. In this study we will develop a new needle guide to assure a shallow angle of approach for internal jugular venous catheterization. The final goal is contributing to safe conduct of ultrasound-guided central venous catheterization.

Commercially available needle guides may increase the success rate but a new needle guide should be made to prevent mechanical complications. Until now, it was thought that increasing the success rate will reduce the complication rate. However, even if the success rate increases, complications still occur. This is because there was a lack of consideration of pitfalls associated with the ultrasound guided procedure.

1.2 Schema

Recruitment of Participants
(First-year residents at Kyorin University Hospital)

↓

Ultrasound-guided CVC Seminar
Lecture for basic skills and simulation training in ultrasound-guided CVC

↓

Demographic data collection from participants
Experience with CVC and ultrasound guidance

↓

Test for ultrasound-guided CVC procedure

1. Free-hand short-axis out-of-plane approach
2. A commercial needle guide for out-of-plane approach
3. A novel shallow angle needle guide

After simulation training, all participants performed each of the 3 approaches using the simulator

↓

Data collection
Posterior vein wall injury rate, success rate, number of needle passes till success, time for the procedure, unanticipated arterial injuries.
A questionnaire to evaluate comfort with the procedure and the preferred procedure using a 5-point Likert scale.

2. Background

Ultrasound guidance during central venous catheterization is associated with high success rates and low mechanical complication rates, and has been recognized as the "gold standard" technique. In our hospital, applicants listen to a didactic lecture for patient safety and an ultrasound-guided central venous catheterization hands-on seminar to obtaining permission to perform CVC. Ultrasound guidance has the benefit of observing the internal jugular vein and the common carotid artery while handling the needle. This benefit leads to a low complication rate compared to the anatomical landmark technique (1). However, sufficient training is required to perform safe central venous catheterization using ultrasound guidance. The operator with poor skills for ultrasound guidance may cause serious complications.

Recently, needle guides have been developed to assist operators during ultrasound guided venous catheterization, in which the needle trajectory is set by the guide while advancing toward the target vein. A novel ultrasound device equipped needle navigation technology was developed based on detecting a magnetic field (2). The needle navigation technology was reported to yield a high success rate and reduce the rate of posterior wall injuries (3). However, our previous study showed that the needle navigation technology did not lead to a reduction of all complications (rate of posterior wall injuries). The study showed that the angle of approach may be related to the rate of posterior wall injuries. Use of a shallow angle of approach using the needle navigation technology decreased the rate of posterior wall injuries, but a steep angle of approach did not. The navigation technology can assist operators to perform the shallow angle of approach. However, ultrasound devices equipped with needle navigation technology are not in widespread use and are not sold in Japan.

3. Objective

We developed a new needle guide to assure a shallow angle of approach for internal jugular venous catheterization. In this study, we evaluate the success rate and rate of posterior vein wall injuries comparing the newly developed needle guide, a conventional commercial needle guide, and the free hand method using a simulated internal jugular vein.

4. Research participants

Participants will be first-year residents who are training in Kyorin University hospital. We conduct a seminar for ultrasound-guided central venous catheterization seminar every year. The participants will be recruited from the seminar as volunteers. Their demographic data will be collected using questionnaires, including prior experience with CVCs, number of cases, experience with ultrasound-guided CVC, and having a CVC license in the institution. Exclusion criteria is previous experience with CVC using a needle guide (the questionnaire was performed after the seminar) (questionnaire is shown in the appendix).

5. Methods
The following three methods are compared:
1. Free-hand short-axis out-of-plane approach (method 1)
2. A commercial needle guide for out-of-plane approach (method 2)
3. A novel shallow angle needle guide (method 3)

After simulation training, all participants perform each of the three approaches using the simulator, and their performance is evaluated. An endoscopic view inside the simulated vessel cannot be seen by participants but is recorded for later review. The video recordings are sequentially numbered, but this number is later randomized by computer to maintain anonymity. The sequence for each participant performing each technique is randomly decided using a random number table. The blinding and allocations are done by a person who does not participate in the trial. The technique used and individual identification are concealed for the evaluation. Two senior physicians who will not participate in the test, observe the recorded videos and evaluate whether the procedure is performed successfully or not.

6. Outcome
The primary outcome of this study is the rate of posterior vein wall injuries. Secondary outcomes include success rate, number of needle passes till success, time for the procedure, and unanticipated arterial injuries. A questionnaire is given to participants to evaluate comfort and the preferred procedure using a 5-point Likert scale (5: very comfortable with the procedure, 1: uncomfortable with the procedure) (questionnaire form in appendix).

7. Number of participants
We plan to include fifty junior residents as participants for the following reason:
A previous study showed that incidence of posterior vin wall injuries was 41% using a steep angle of approach and 9 % with a shallow angle (4). The sample size required for 80% power at $\alpha = 0.05$ was estimated to be thirty-four participants. Fifty participants are included to account for exclusions and being a preliminary study.

8. Simulator, research environment
The simulated internal jugular vein is connected to a water tank through a tube to maintain pressure at 10 cm H_2O, which is monitored by a pressure transducer. The simulated carotid artery is pressurized to 50 cm H_20 using the same method. An endoscope is inserted into the simulated internal jugular vein and an interior view of the vessel is recorded during the procedure. After each procedure, the participant waits at least 15 minutes as a washout period before performing the next procedure.

9. Equipment

① Endoscopic video system (owned by the Department of Anesthesiology)
② Commercial needle guide AccuSITE™ (Civco Co., USA) (owned by the department)
③ Novel needle guide with a shallow angle of approach (owned by the department). The needle guide was created using 3D modeling software, 3D Builder (Microsoft Co., USA), and was made of nylon using a 3D print service (DMM.make Co., Japan) (Figures 1, 2).

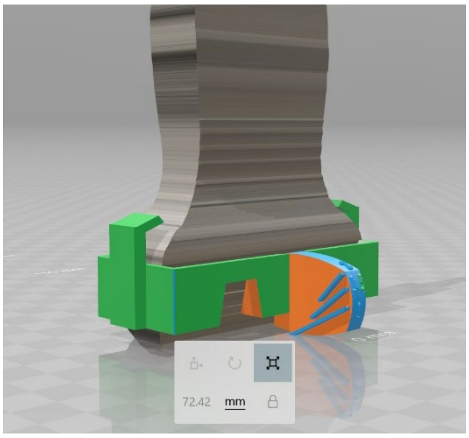

Fig 1. 3D picture of needle guide design

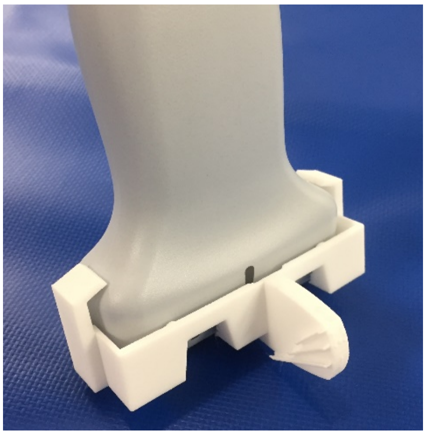
Fig 2. Needle guide

④ The ultrasound machine is the EDGE HFL50 or HFL38 (FUJIFILM SonoSite, Inc., USA) (owned by the department).
⑤ Simulator (owned by the department)

10. Research period
After approval by the Ethics Committee until August 31, 2020

11. Institution and study site
OR Center and Clinical simulation laboratory in Kyorin University Hospital

12. Expected results (research hypothesis)
Posterior wall puncture rate when operators use the novel needle guide with a shallow angle of approach will be significantly lower than when using the two other techniques.

13.Data anonymization: The data will be collected using an identification number instead of participant names and create a correspondence table for managing the data.

14. Ethical considerations: This is not a clinical study, but a simulator study. Participants are recruited as volunteers. Written informed consent will be obtained from all participants. We are disclosing research information (in the announcement form “Call for study participants”) and participant rights (being able to stop participating at any time). The advantage to the participants is taking the seminar for

free. There are no patients involved in this study.

15.Expenses
① Disposable bracket for a commercial needle guide (¥3000 each, 12 /box)
② 3D printing service (¥3000 each) 10 guides
③ Vein simulator UGP GEL (¥9000) 10

16.Research team
Principal investigator: Kunitaro Watanabe (Department of Anesthesiology, Fixed term assistant professor, postgraduate student)
Research director: Joho Tokumine (Department of Anesthesiology, Professor)
Co-investigator: Kumi Moriyama (Department of Anesthesiology, Lecturer)

17.References
1. Brass P, Hellmich M, Kolodziej L, Schick G, Smith AF. Ultrasound guidance versus anatomical landmarks for internal jugular vein catheterization. Cochrane Database Syst Rev. 2015;1:CD006962. doi: 10.1002/14651858.CD006962.pub2.

2. Gadsden J, Latmore M, Levine DM. Evaluation of the eZono 4000 with eZGuide for ultrasound-guided procedures. Expert Rev Med Devices. 2015 ;12:251-61. doi: 10.1586/17434440.2015.995095. Epub 2014 Dec 29.

3. Auyong DB, Yuan SC, Rymer AN, Green CL, Hanson NA. A randomized crossover study comparing a novel needle guidance technology for simulated internal jugular vein cannulation. Anesthesiology. 2015;123:535-41. doi: 10.1097/ALN.0000000000000759.
