# Supplementary material for: Shallow-angle needle guide for ultrasound-guided internal jugular venous catheterization: A randomized controlled crossover simulation study (CONSORT)
Source: PLoS One. 2020 Jun 30;15(6):e0235519. doi: 10.1371/journal.pone.0235519 (PMC7326219; doi:10.1371/journal.pone.0235519)
Supplement: Attachment — Submitted filename: Response to Reviewers.docx [file pone.0235519.s004.docx]

**Response to Reviewers**

Thank you very much for reviewing our paper. We answered your questions a point-by-point fashion below.

**Journal Requirements:**

When submitting your revision, we need you to address these additional requirements.

1. Please ensure that your manuscript meets PLOS ONE's style requirements, including those for file naming.

Thank you very much. We revised our manuscript to meet PLOS ONE's style requirements.

2. Thank you for stating the following financial disclosure:

'The funders had no role in study design, data collection and analysis, decision to publish, or preparation of the manuscript.'

At this time, please address the following queries:

We have not received funding from any organization for this study. We stated this in the cover letter.

*The authors received no specific funding for this work.*

3. Please amend either the title on the online submission form (via Edit Submission) or the title in the manuscript so that they are identical.

We corrected the title of the revised manuscript.

*Shallow-angle needle guide for ultrasound-guided internal jugular venous catheterization:*

*A randomized controlled crossover simulation study (CONSORT)*

4. Please ensure that you refer to Figure 5 in your text as, if accepted, production will need this reference to link the reader to the figure.

We made a mistake in numbering the figures. We renumbered them as follows.

P6 L66

*The needle guide was made of nylon using a 3D print service (DMM.make Co., Japan) (Fig 2).*

P7 L88

*This study was planned as a randomized crossover-controlled trial. Participants received a didactic lecture to learn basic skills in ultrasound-guided central venous catherization. Instructors demonstrated how to perform three approaches, including free-hand (P-free) (Fig 3), using a commercially available needle guide (P-com) (Fig 4), and using the newly developed needle guide (P-sha) (Fig 5).*

**Reviewers' comments:**

**Reviewer's Responses to Questions**

Comments to the Author

Thank you very much for your comments.

Reviewer #1: Interesting paper

Some issues.

1) posterior vein wall injuries should be defined

Thank you for your advice. We defined posterior vein wall injuries as follows.

P9 L107

*A posterior vein wall injury was defined as obvious penetration of the posterior vein wall by the needle on visual inspection*.

2) shallow should be better defined

Thank you for your advice. We defined it as follows.

P6 L69

*In this study we defined a shallow angle as under 30 º.*

3) the present is a randomized controlled trial; this should be better specified in the title

Thank you for your advice. We corrected the title.

Shallow-angle needle guide for ultrasound-guided internal jugular venous catheterization:

A randomized *controlled* crossover simulation study (CONSORT)

4) 5) sample size calculation is missing due to reduced sample size normal distribution should be tested for.

Thank you for your comment. We described the calculation of sample size in Statistical Analysis.

P6 L58

*Sample size was calculated using a previous study [5] which is described below in the statistical analysis section.*

P9 L115

*Sample size calculation*

*A previous study showed that incidence of posterior vin wall injury is 41% using a steep angle of approach and 9 % with a shallow angle [5]. Based on this data the sample size required for 80% power at ɑ = 0.05 was estimated to be thirty-four participants and we planned to include thirty-five junior residents as participants. Sample size was calculated with EZR using R commander, version 1.32 (Saitama Medical Center, Jichi Medical University, Saitama, Japan) [8].*
